# Association of Adverse Childhood Experiences Including Low Household Income and Peer Isolation With Obesity Among Japanese Adolescents: Results From A-CHILD Study

**DOI:** 10.3389/fpubh.2022.754765

**Published:** 2022-04-05

**Authors:** Satomi Doi, Aya Isumi, Takeo Fujiwara

**Affiliations:** ^1^Department of Global Health Promotion, Tokyo Medical and Dental University (TMDU), Tokyo, Japan; ^2^Research Fellow of Japan Society for the Promotion of Science, Tokyo, Japan

**Keywords:** adolescent, adverse childhood experience, low household income, obesity, peer isolation

## Abstract

**Background:**

Adverse childhood experience (ACE) is a major risk factor for obesity in both adults and adolescents. Although, arguably, peer isolation and low household income could be conceived as of ACEs, few studies have included these experiences as ACEs.

**Objectives:**

This study aims to examine whether ACEs, including peer isolation and low household income, are associated with obesity in adolescents.

**Methods:**

We used pooled data from the Adachi Child Health Impact of Living Difficulty (A-CHILD) study in 2016 and 2018, which is a school-based cross-sectional study in Adachi City, Tokyo, Japan, *N* = 6,946, 4th (9–10 years old), 6th (11–12 years old), and 8th (13–14 years old) grades. Among the eight items of ACEs, adolescents assessed one item, including peer isolation, and their caregivers assessed seven other items using questionnaires. The adolescents' body mass index (BMI) was measured in school health checkups and calculated to fit the World Health Organization (WHO) standards. Multinomial logistic regression was applied to investigate the association of the cumulative ACEs and each type of ACE with BMI, in which the study was conducted in 2020.

**Results:**

The number of ACEs was not associated with overweight or obesity among adolescents after adjusting for covariates. As for each type of ACE, single parenthood and low household income showed a significant independent association with obesity.

**Conclusions:**

The number of ACEs was not associated with overweight or obesity in Japanese adolescents, while single parenthood and low household income showed a significant positive association with obesity. Further longitudinal studies are needed to replicate this association among adolescents.

## Introduction

Overweight and obesity among adolescents are worldwide public health issues (1), which lead to adverse health outcomes in adolescents, such as poor lung function and asthma (2, 3), psychological and behavioral problems, and (4) poor health-related quality of life (5). Furthermore, obesity in adolescents is associated with an increased risk of adverse health outcomes in adulthood, including severe obesity (6), diabetes (7), cardiovascular disease (8), and psychological problems (9).

Adverse childhood experiences (ACEs), including parental loss; household dysfunction, such as parental psychiatric disorders; and child maltreatment, such as physical abuse and neglect before the age of 18 years (10), are some of the well-established risk factors for overweight and obesity in both adults and adolescents (11). A longitudinal cohort study found that adolescents aged 15 years with many ACEs before 11 years of age were more likely to be overweight than those without ACEs (12). Another population-based prospective cohort study has shown that ACEs before 9 years of age are related to overweight and obesity at 13 years of age, which is the age of early adolescence (13). However, knowledge about the association between ACEs, overweight, and obesity among adolescents in Asian cultures is scarce because overweight and obesity are less prevalent than that in Western countries (14–16). Although other studies have shown similar associations between ACEs and health among older adults (17), ACEs may have differential impacts on overweight and obesity in Japan because psychical abuse is more common in Asian countries than in Western countries (18).

Despite these findings, a meta-analysis that examined the association between ACEs and overweight and obesity in childhood, including adolescents (19), suggested that further studies are needed due to heterogeneity in the measurement of ACEs. Peer isolation and low household income, in particular, can be especially influential ACEs for adolescents, as they have a significant role in determining the psychological and physical health not only in adolescence but also in adulthood. Moreover, these exposure categories were recently added to the original ACE scale (20). Peer isolation is an important part of adolescence (21) because of the shift in social support from family members to friends (22). Hall-Lande et al. (22) indicated that peer relationships nurture group alliances to provide psychological support and a sense of belonging at a time when adolescents develop their personal identity (23), social competence, and self-worth. Peer isolation in adolescence, which means the lack of the above benefits of close peer relationships, is associated with poor psychological health (22), including smoking behavior (24) in adolescence. Additionally, peer isolation in adolescence induces poor psychological health, poor health behaviors, increased metabolic syndrome (25), and the risk of premature mortality (26) in adulthood.

A low household income is considered an important ACE (20). However, there is controversy regarding whether low household income in childhood itself is considered as ACE (27) because low household income can be a root cause of ACEs (28). Contrarily, previous studies have found that the association between ACEs and health outcomes remains even after adjusting for socioeconomic positions in childhood (29, 30). Furthermore, socioeconomic deprivation is independently associated with a high risk of overweight and obesity among adolescents after adjustment of ACEs (31). These findings may indicate that low household income may have a direct impact on adolescent health, independent of ACEs. Thus, in this study, low household income was considered as ACE, and the impact of low household income on body mass index (BMI) was examined.

In this study, we examined whether the accumulation of ACEs, including peer isolation and low household income, is associated with BMI in adolescents. Furthermore, we assessed the association between each type of ACE and BMI.

## Methods

### Study Sample

Pooled data were obtained from the Adachi Child Health Impact of Living Difficulty (A-CHILD) study in 2016 and 2018, which is a school-based cross-sectional study that examines the living environment and health of elementary and junior high school students and their caregivers in Adachi City, Tokyo, Japan. Details of the A-CHILD study are reported in the protocol paper (32).

**Figure 1:**
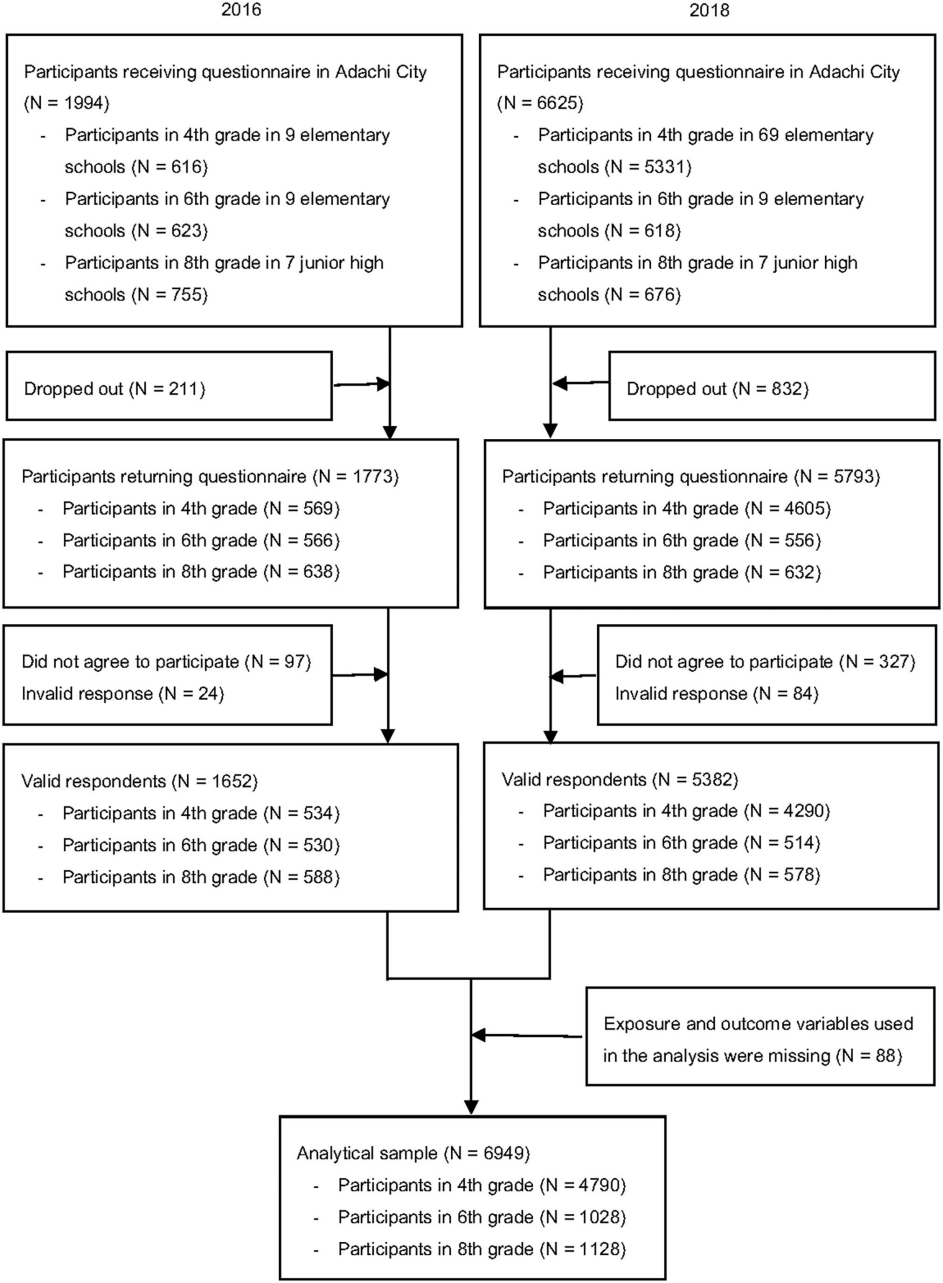
Participants flow chart.

We used data from children aged 9–10 years, such as 4th grade, in 9 representative elementary schools collected in 2016, and all 69 elementary schools surveyed in 2018 (*N* = 4,790), 11–12 years, such as 6th grade in 9 representative elementary schools (*N* = 1,028), and 13–14 years, such as 8th grade in 7 representative junior high schools (*N* = 1,128). The cross-sectional data were pooled to maximize the sample size. Adolescents brought anonymous self-reported questionnaires with unique identifications (IDs) home to their caregivers. The questionnaires were distributed to 2,014 and 6,605 adolescents in 2016 and 2018, respectively. The number of adolescents who returned both adolescent and caregiver questionnaires with valid responses, such as with at least one response, obtained informed consent, and linked with school health checkup, was 1,652 (response rate = 82.8%) in 2016 and 5,382 (response rate = 81.2%) in 2018. Among the valid responses, we excluded participants who missed exposure and outcome variables in this study, including the ACEs and BMI. Finally, our analytical sample comprised 6,949 adolescents (Figure 1).

### Measures

Adverse childhood experiences consist of eight items, namely, single parenthood, parental history of psychiatric disorders, physical and/or psychological abuse, witnessing domestic violence, neglect, peer isolation, and low household income (20). Peer isolation was self-assessed by the adolescents, and the other seven items were assessed by their caregivers using questionnaires. Single parenthood was assessed by the question of “living with family members.” Parental history of psychiatric disorders was assessed by two questions, namely, maternal history of psychiatric disorders and paternal history of psychiatric disorders. As for low household income, the caregivers were asked about their annual household income (<500,000 yen, 500,000 to <1,000,000 yen, 1,000,000 to <2,000,000 yen, 2,000,000 to <3,000,000 yen, 3,000,000 to <4,000,000 yen, 4,000,000 to <5,000,000 yen, 5,000,000 to <6,000,000 yen, 6,000,000 to <7,500,000 yen, 7,500,000 to <10,000,000 yen, 10,000,000+ yen, or unknown, where 1,000,000 yen is equivalent to US$ 10,000). The responses were dichotomized with “ <500,000 yen,” “500,000 to <1,000,000 yen,” “1,000,000 to <2,000,000 yen,” and “2,000,000 to <3,000,000 yen” equated to a “yes” response.

Physical abuse, psychological abuse, witnessing domestic violence, and neglect were assessed using seven questions on a scale of 1 = “often,” 2 = “sometimes,” 3 = “rarely,” and 4 = “not at all.” Physical abuse was assessed with two questions, namely, “hit the child's body (buttocks, hand, head, or face),” in which the responses were dichotomized with “often” equated to a “yes” response, and “beat the child,” in which the responses were dichotomized with “rarely,” “sometimes,” or “often” equated to a “yes” response. We coded physical abuse as 1 (yes) when either item was classified as “yes.” Psychological abuse was assessed according to two inquiries, namely, “yell at the child,” in which the responses were dichotomized with “often” equated to a “yes” response, and “insult the child repeatedly,” in which the responses were dichotomized with “sometimes” or “often” both of which equated to a “yes” response. We coded psychological abuse as 1 (yes) when either question was classified as “yes”. We coded psychological abuse as 1 (yes) when either item was classified as “yes.” Witness of domestic violence was assessed as “have a big fight in front of the child,” in which the responses were dichotomized with “sometimes” or “often” which were equated to a “yes” response. Neglect was assessed by: “shut the child outside” and “do not feed the child,” in which the responses were dichotomized with “rarely,” “sometimes” or “often” which were equated to a “yes” yes response. We coded neglect as 1 (yes) when either experience was classified as “yes.” These coding guidelines were developed in a previous study (33).

The adolescents' BMI was calculated to fit the World Health Organization (WHO) standards (34) using height and weight measured during school health checkups. BMI was further categorized as underweight (< -1 SD), normal (≥-1 SD, <1 SD), overweight (≥1 SD, <2 SD), and obese (≥2 SD).

Covariates, such as child's sex (“male” or “female”), grade, and maternal age, were measured using the questionnaire. Additionally, other environmental factors, including maternal and paternal BMI calculated using their height and weight (<18.5, 18.5 <25, 25 <30, or 30+), child's screen time on weekdays per day (“0 min,” “30 min <2.5 h,” or “3 h +”), physical activity on weekdays (“not at all,” or “sometimes or more”), and frequency of having breakfast (“every day,” “sometimes,” or “not at all”), were measured using the questionnaire.

### Ethics

This study was approved by the Ethics Committee of the Tokyo Medical and Dental University (M2016-284).

### Statistical Analysis

First, the correlation coefficients among the characteristics, exposure, and outcome variables were estimated. Second, multinomial logistic regression was applied to investigate the association of cumulative ACEs and each type of ACE with BMI categories. In the analyses, we used a cumulative ACE total score (0, 1, 2, or, 3+), which was calculated using all eight items for categorical variables and each type of ACE. Third, linear regression analysis was used for BMI z scores. All analyses were weighted by the number of participants in each grade. Model 1 included maternal age (<30, 30–34, 35–39, 40–44, and 45+ years), child's sex, grade, year of survey, and school in Adachi City, which were decided based on a previous study (35). Furthermore, Model 2 added parental obesity, defined as either mother or father with obesity, into Model 1. There were missing data in only maternal age and parental obesity, which were treated as dummy variables. All analyses were conducted in 2020 using the STATA version 15.0 SE.

## Results

Table 1 shows the distribution of characteristics by ACE score among the participants. Among all the participants, 60.2% had no ACEs, 19.4% had one ACE, 13.9% had two ACEs, and 6.5% had three or more ACEs. Adolescents with three or more ACEs were more likely to be male and young. Mothers who had children with three or more ACEs were more likely to be young. Regarding BMI, 13.4% were overweight, and 5.3% were obese. In terms of other environmental factors, mothers who had children with three or more ACEs were more likely to be obese, such as with a BMI <30, and fathers who had children with a high number of ACE were less likely to have a normal BMI, such as with a BMI of 18.5 <25. Furthermore, children with three or more ACEs were more likely to report no screen time or excessive screen time, such as 3 h or more per day, report less physical activity, and report not having breakfast every day.

**Table 1:** Characteristics of the sample (*N* = 6,946).

		**Total**		**Number of adverse childhood experiences**								***p* for χ2 test**
				**0 (*****N*** **=** **4,093; 60.2%)**		**1 (*****N*** **=** **1,318; 19.4%)**		**2 (*****N*** **=** **946; 13.9%)**		**3+** **(*****N*** **=** **442; 6.5%)**		
Child's sex	Male	3,474	50.0	1,917	55.2	624	18.0	618	17.8	315	9.1	<0.001
	Female	3,472	50.0	2,227	64.1	710	20.4	376	10.8	159	4.6	
Grade	4^th^	4,790	69.0	2,771	57.8	908	19.0	729	15.2	382	8.0	<0.001
	6^th^	1,028	14.8	663	64.5	192	18.7	124	12.1	49	4.8	
	8^th^	1,128	16.2	710	62.9	234	20.7	141	12.5	43	3.8	
Maternal age	<30	54	0.8	17	31.5	18	33.3	7	13.0	12	22.2	<0.001
	30–34	586	8.4	272	46.4	150	25.6	101	17.2	63	10.8	
	35–39	1,548	22.3	891	57.6	318	20.5	219	14.1	120	7.8	
	40–44	2,502	36.00	1,580	63.1	446	17.8	329	13.1	147	5.9	
	45+	2,008	28.9	1,219	60.7	356	17.7	310	15.4	123	6.1	
	Missing	248	3.6	165	66.5	46	18.5	28	11.3	9	3.6	
BMI category	Normal (−1SD- <1SD)	4,438	63.9	2,709	61.0	831	18.7	600	13.5	298	6.7	0.003
	Underweight (< -1SD)	1,212	17.4	723	59.6	246	20.3	175	14.4	68	5.6	
	Overweight (1SD < -2SD)	928	13.4	510	55.8	184	19.8	150	16.2	76	8.2	
	Obesity (2SD+)	368	5.3	194	52.7	73	19.8	69	18.7	32	8.7	
Maternal BMI category	<18.5	766	11.0	440	10.6	164	12.3	108	10.9	54	11.4	0.001
	18.5 <25	4,651	67.0	2,809	67.8	890	66.7	663	66.7	289	61.0	
	25 <30	695	10.0	382	9.2	128	9.6	113	11.4	71	15.2	
	30+	131	1.9	70	1.7	25	1.9	19	1.9	17	3.6	
	Missing	703	10.1	443	10.7	127	9.1	91	9.1	42	8.9	
Parental BMI category	<18.5	95	1.4	50	1.2	15	1.1	20	2.0	10	2.1	<0.001
	18.5 <25	3,667	52.8	2,375	57.3	612	45.9	486	48.9	194	40.9	
	25 <30	1,505	21.7	943	22.8	254	19.0	217	21.8	91	19.2	
	30+	287	4.1	163	3.9	60	4.5	41	4.1	23	4.8	
	Missing	1,392	20.0	613	14.8	393	29.5	230	23.1	156	32.9	
Screen time (weekdays, per day)	0m	618	8.9	344	8.3	129	9.7	90	9.0	55	11.6	0.005
	30 m <2.5 h	3,993	57.5	2,438	58.8	753	56.4	549	55.2	253	53.4	
	3h+	1,107	15.9	609	14.7	231	17.3	176	17.7	91	19.2	
	Missing	1,228	17.7	753	18.2	221	16.6	179	18.0	75	15.8	
Physical activity (weekdays)	Not at all	853	12.3	456	11.0	154	11.5	158	15.9	84	17.7	<0.001
	Sometimes or more	5,546	79.8	3,341	80.6	1,081	81.0	764	76.9	360	75.9	
	Missing	548	7.9	347	8.4	99	7.4	72	7.2	30	6.3	
Having breakfast	Everyday	6,114	88.0	3,766	90.9	1,108	83.1	858	86.3	382	80.6	<0.001
	Sometimes	579	8.3	261	6.3	163	12.2	91	9.1	64	13.5	
	Not at all	191	2.7	43	2.2	43	3.2	35	3.5	22	4.6	
	Missing	62	0.9	20	0.6	20	1.5	10	1.0	6	1.3	

The distribution of each type of ACE by grade is presented in Table S1. Among the total participants, over 10% had experienced single parenthood, peer isolation, and low household income. In each grade, approximately 10% of the adolescents experienced these three types of ACEs. Table S1 also shows the relationship between each type of ACE. We found a high comorbidity of ACEs. Single-parent households also had low household incomes in 61.8% of the adolescents. Furthermore, 42.0% of the adolescents who experienced psychological abuse from their parents also experienced physical abuse from their parents.

The distribution of BMI categories by grade is shown in Table S2. Among all participants, 63.9% of the adolescents were in the normal range, 17.4% were underweight, 13.4% were overweight, and 5.3% were obese. The proportion of overweight and obesity decreased as adolescent age increased (overweight: 4th = 14.3%, 6th = 12.0%, 8th = 10.6%: obesity: 4th = 5.7%, 6th = 5.1%, and 8th grade = 3.7%).

After estimating the correlation coefficients (Table S3), we conducted a multinomial logistic regression (Table 2). In the crude model, adolescents who experienced two ACEs were 32% more likely to be overweight (relative risk ratio [RRR] = 1.32; 95% confidence interval [CI] = 1.06–1.64) and 55% more likely to be obese (RRR = 1.55; 95% CI = 1.13–2.12) compared with those without ACEs. However, three or more ACEs showed no significant associations, which could be due to a lack of power. In Model 1, in which maternal age, child's sex, grade, year of survey, and schools in Adachi City were added, the associations were not significant (overweight: RRR = 1.24; 95% CI = 0.98–1.55; obesity: RRR = 1.28; 95% CI = 0.92–1.78). In Model 2, in which parental obesity was added, we also found no significant association (overweight: RRR = 1.22; 95% CI = 0.97–1.54; obesity: RRR = 1.29; 95% CI = 0.92–1.80). As for each type of ACE, only low household income (RRR = 1.31; 95% CI = 1.04–1.66) was associated with overweight, which remained in Model 1 (RRR = 1.32; 95% CI = 1.03–1.70) and Model 2 (RRR = 1.34; 95% CI = 1.05–1.72). Single parenthood (RRR = 1.65; 95% CI = 1.23–2.20), peer isolation (RRR = 1.50; 95% CI = 1.09–1.98), and low household income (RRR = 1.65; 95% CI = 1.19–2.29) were significantly associated with obesity in the crude model. In Models 1 and 2, the significant association of single parenthood (Model 1: RRR = 1.60; 95% CI = 1.18–2.18; Model 2: RRR = 1.72; 95% CI = 1.23–2.50) and low household income (Model 1: RRR = 1.64; 95% CI = 1.16–2.33; Model 2: RRR = 1.75; 95% CI = 1.23–2.50) with obesity remained.

**Table 2:** Results of multinomial logistic regression to examine the associations between ACEs and BMI (*N* = 6,946).

		**Underweight**			**Overweight**			**Obesity**		
		**Crude**	**Model 1**	**Model 2**	**Crude**	**Model 1**	**Model 2**	**Crude**	**Model 1**	**Model 2**
		**RRR (95% CI)**	**RRR (95% CI)**	**RRR (95% CI)**	**RRR (95% CI)**	**RRR (95% CI)**	**RRR (95% CI)**	**RRR (95% CI)**	**RRR (95% CI)**	**RRR (95% CI)**
ACE total score (0–8)	0	Ref	Ref	Ref	Ref	Ref	Ref	Ref	Ref	Ref
	1	1.05 (0.87–1.26)	1.06 (0.88–1.28)	1.06 (0.88–1.28)	1.20 (0.98–1.47)	1.18 (0.96–1.44)	1.17 (0.95–1.44)	1.17 (0.86–1.59)	1.12 (0.82–1.54)	1.12 (0.81–1.54)
	2	1.11 (0.91–1.37)	1.17 (0.95–1.45)	1.17 (0.95–1.45)	**1.32 (1.06−1.64)**	1.24 (0.98–1.55)	1.22 (0.97–1.54)	**1.55 (1.13–2.12)**	1.28 (0.92–1.78)	1.29 (0.92–1.80)
	3+	0.90 (0.67–1.21)	0.91 (0.67–1.23)	0.91 (0.67–1.24)	1.23 (0.92–1.65)	1.15 (0.85–1.55)	1.14 (0.84–1.54)	1.35 (0.89–2.06)	1.06 (0.68–1.66)	1.04 (0.66–1.62)
Single parenthood	No	Ref	Ref	Ref	Ref	Ref	Ref	Ref	Ref	Ref
	Yes	0.87 (0.70–1.08)	0.84 (0.67–1.06)	0.82 (0.65–1.03)	1.21 (0.98–1.50)	1.20 (0.96–1.50)	1.22 (0.97–1.52)	**1.65 (1.23–2.20)**	**1.60 (1.18–2.18)**	**1.72 (1.26–2.36)**
Parental history of psychiatric disorders	No	Ref	Ref	Ref	Ref	Ref	Ref	Ref	Ref	Ref
	Yes	0.95 (0.72–1.24)	0.94 (0.72–1.24)	00.95 (0.72–1.26)	1.00 (0.75–1.34)	0.99 (0.74–1.34)	0.99 (0.74–1.32)	0.90 (0.57–1.41)	0.90 (0.57–1.44)	0.85 (0.53–1.38)
Physical abuse from parents (hit, slap)	No	Ref	Ref	Ref	Ref	Ref	Ref	Ref	Ref	Ref
	Yes	1.16 (0.91–1.47)	1.20 (0.94–1.53)	1.21 (0.95–1.55)	1.25 (0.97–1.61)	1.21 (0.94–1.57)	1.19 (0.92–1.55)	1.21 (0.83–1.76)	1.06 (0.71–1.57)	1.00 (0.67–1.49)
Psychological abuse from parents (verb)	No	Ref	Ref	Ref	Ref	Ref	Ref	Ref	Ref	Ref
	Yes	1.14 (0.82–1.58)	1.20 (0.86–1.67)	1.23 (0.88–1.72)	0.77 (0.51–1.15)	0.76 (0.50–1.14)	0.75 (0.49–1.13)	1.33 (0.80–2.19)	1.22 (0.72–2.10)	1.17 (0.69–2.01)
Witness of domestic violence between parents	No	Ref	Ref	Ref	Ref	Ref	Ref	Ref	Ref	Ref
	Yes	1.06 (0.75–1.50)	1.05 (0.74–1.48)	1.05 (0.74–1.49)	0.98 (0.67–1.44)	1.02 (0.69–1.52)	1.01 (0.68–1.50)	1.09 (0.63–1.88)	1.12 (0.63–1.99)	1.06 (0.60–1.88)
Neglect from parents (out, unfed)	No	Ref	Ref	Ref	Ref	Ref	Ref	Ref	Ref	Ref
	Yes	1.05 (0.82–1.35)	1.06 (0.82–1.37)	1.06 (0.82–1.37)	1.06 (0.81–1.39)	1.03 (0.78–1.35)	1.02 (0.78–1.35)	0.86 (0.56–1.34)	0.76 (0.49–1.19)	0.81 (0.52–1.27)
Peer isolation	No	Ref	Ref	Ref	Ref	Ref	Ref	Ref	Ref	Ref
	Yes	0.99 (1.08–1.56)	1.03 (0.83–1.28)	1.03 (0.83–1.29)	1.23 (0.99–1.52)	1.13 (0.90–1.41)	1.12 (0.89–1.40)	**1.50 (1.09–1.98)**	1.15 (0.83–1.57)	1.14 (0.82–1.57)
Low household income (<3,000,000)	No	Ref	Ref	Ref	Ref	Ref	Ref	Ref	Ref	Ref
	Yes	0.96 (0.76–1.22)	0.94 (0.74–1.20)	0.93 (0.72–1.18)	**1.31 (1.04–1.66)**	**1.32 (1.03–1.70)**	**1.34 (1.05–1.72)**	**1.65 (1.19–2.29)**	**1.64 (1.16–2.33)**	**1.75 (1.23–2.50)**

*All analyses were weighted for grade. Model 1 included maternal age, child's sex, grade, year of survey, and schools in Adachi City. Model 2 added parental obesity into Model 1*.

*ACE, adverse childhood experience; BMI, body mass index; RRR, relative risk ratio; 95% CI, 95% confidence interval*.

*Bold values indicate p <0.001*.

Table S4 shows the results of linear regression analysis. Adolescents who experienced two ACEs and three or more ACEs showed a higher BMI z score (2 ACEs: β = 0.13, 95% CI = 0.04–0.22; 3 or more ACEs: β = 0.12, 95% CI = 0.01–0.24) compared to those without ACEs in the crude model. The adjusted model showed no significant association. For each type of ACE, single parenthood (β = 0.15, 95% CI = 0.07–0.24), peer isolation (β = 0.13, 95% CI = 0.04–0.22), and low household income (β = 0.14, 95% CI = 0.05–0.24) showed a high BMI z score in the crude model. In terms of single parenthood and low household income, the significant association remained in both Model 1 (single parenthood: β = 0.14, 95% CI = 0.05–0.23; low household income: β = 0.14, 95% CI = 0.04–0.24) and Model 2 (single parenthood: β = 0.16, 95% CI = 0.07–0.25; low household income: β = 0.15, 95% CI = 0.05–0.25).

## Discussion

In this study, we examined the association of ACEs, including peer isolation and low household income, with BMI in Japanese adolescents. We found that the number of ACEs was not associated with being overweight or obese after adjusting for covariates. However, single parenthood and low household income are associated with obesity. The childhood experiences of single parenthood and low household income might predict obesity in Japanese adolescents.

As for the accumulation of ACEs, the significant association of two ACEs with overweight and obesity became nonsignificant after adjusting for maternal age, child's sex, grade, year conducted a survey, school, and parental obesity. Although some studies have found an association between the accumulation of ACEs and increased BMI in adolescents (12, 13, 19), other studies have shown no significant association between child abuse and increased BMI (36, 37). Sokol et al. (37) who examined the association between child abuse and BMI across adolescence found that adolescents who experienced abuse, who did not experience physical abuse, and who experienced both physical abuse and neglect all exhibited stable BMIs. However, adolescents who experienced sexual abuse and co-occurring physical abuse and neglect showed a rapid increase in BMI. In this study, we did not assess sexual abuse or the combination of each type of child abuse and neglect. Further study is needed to examine the impact of sexual abuse and co-occurring child abuse on BMI in adolescents.

We found a significant positive association between single parenthood and overweight, which is consistent with previous studies (12, 13, 19). In previous studies, single parenthood and low household income were strong predictors of obesity in children (38, 39). Furthermore, adolescents living in single-parent households were 60% more likely to be obese, and adolescents living in poverty were 32% more likely to be overweight and 63% more likely to be obese. Single parenthood and low household income overlapped by ~60% (see Table S1), which indicates that the pathways of single parenthood and low household income to overweight may be similar. Single-parent families are more likely to face financial problems and are less likely to have time and capacity to support their children's healthy eating and physical activity. Similarly, parents with low incomes were less likely to have money and time to prepare healthy food for their children (40), which was confirmed in Japan (41). Moreover, adolescents living in poverty are more likely to engage in unhealthy behaviors, such as irregular exercise and excessive screen time (42).

Peer isolation was found to be associated with obesity in the crude model but was not significant in the adjusted model. Nonetheless, peer isolation is a remarkable childhood experience because peer isolation in adolescence is associated with not only obesity but also other adverse health outcomes in adulthood (43). The association between peer isolation and obesity in adolescents can be explained by two possible pathways. First, peer isolation may induce a critical state for children by biological changes. Previous studies have found that social isolation is associated with a high cholesterol response to stress (24), elevated blood pressure, fibrinogen, and C-reactive protein (44, 45), which increases the risk of diseases such as cardiovascular disease (46). Although these studies included participants who were in adulthood and old age, biological changes related to overweight and obesity due to peer isolation may also appear in children. Second, social isolation is associated with low levels of physical activity, which leads to overweight and obesity. Among older adults, the association between social isolation and low levels of physical activity is well established (47). The study examining this association in adolescents is growing (48, 49). According to the results of the adjusted model, further study to examine the different impacts of peer isolation by children's age and sex is needed.

According to our findings, the association of single parenthood and low household income with overweight and obesity already appeared among adolescents. Even though there is a controversy regarding whether socioeconomic positions in childhood, such as single parenthood and low household income, are considered as ACE (27), our results indicate the need to focus not only on child maltreatment but also on socioeconomic positions to prevent overweight and obesity among adolescents. Additionally, our findings may contribute to policymaking, such as a combination approach of providing parenting skills for parents, establishing a child's healthy lifestyle, and providing financial support for single-parent families and parents living in poverty. These policies may prevent not only overweight and obesity but also child maltreatment because socioeconomic positions in childhood can be a root cause of child maltreatment (28).

This study has several limitations. First, as this is a cross-sectional study, we cannot indicate the causal relationship between ACE and BMI, as adolescents with high BMI might be more likely to be isolated from peers (50). Additionally, there might be recall bias because of the retrospective reporting of ACEs. Further longitudinal studies are needed to examine the causal relationship between ACE and BMI in adolescents. Second, there might be unmeasured confounders in this study, such as maternal education level, parental employment status, and child birth weight (37). Third, sampling bias related to responses to the questionnaire might exist, even though the response rate in this study was high. Adolescents who had experiences of peer isolation and caregivers who lived in poverty had mental health problems or reported child maltreatment might be less likely to respond to the questionnaire. Thus, our results may have been underestimated by this selection bias. Fourth, the ACE did not include the experience of sexual abuse in this study. Sexual abuse is considered to be a sensitive issue, and its prevalence is low, approximately 0.5% (51), but may still lead to a low response rate.

## Conclusion

We found no association between the number of ACEs and being overweight. Specific ACEs, single parenthood, and low household income showed a significant risk of being overweight among Japanese adolescents. To date, interventions for single-parent families and parents living in poverty have been developed to prevent childhood overweight and obesity (52, 53). However, families with low socioeconomic status are less likely to participate in these prevention interventions (54). Thus, population approach programs to prevent overweight and obesity in adolescents need to be developed. Further longitudinal studies are needed to replicate the causal relationship between ACE and BMI in adolescents and to develop a population approach program to prevent overweight and obesity in adolescents.

## Data Availability Statement

The datasets presented in this article are not readily available because the data presented in this study are available on request from the corresponding author. The data are not publicly available due to ethical restrictions. Requests to access the datasets should be directed to fujiwara.hlth@tmd.ac.jp.

## Ethics Statement

The studies involving human participants were reviewed and approved by Ethics Committee of the Tokyo Medical and Dental University. Written informed consent to participate in this study was provided by the participants' legal guardian/next of kin.

## Author Contributions

TF designed the study, supervised the analysis, and critically revised the manuscript. SD conducted literature searches, provided summaries of previous research studies, conducted the statistical analysis, and wrote the first draft of the manuscript. AI developed the study method. All authors have contributed to and approved the final manuscript.

## Funding

This study was supported by the Health Labor Sciences Research Grant, Comprehensive Research on Lifestyle Disease from the Japanese Ministry of Health, Labor and Welfare (H27-Jyunkankito-ippan-002), Research on the Policy Planning and Evaluation from the Japanese Ministry of Health, Labor and Welfare (H29-Seisaku-Shitei-004), Innovative Research Program on Suicide Countermeasures (IRPSC), and Grants-in-Aid for Scientific Research from the Japan Society for the Promotion of Science (JSPS KAKENHI Grant Number 16H03276, 16K21669, and 18K13318), St. Luke's Life Science Institute Grants, and the Japan Health Foundation Grants.

## Conflict of Interest

The authors declare that the research was conducted in the absence of any commercial or financial relationships that could be construed as a potential conflict of interest.

## Publisher's Note

All claims expressed in this article are solely those of the authors and do not necessarily represent those of their affiliated organizations, or those of the publisher, the editors and the reviewers. Any product that may be evaluated in this article, or claim that may be made by its manufacturer, is not guaranteed or endorsed by the publisher.
