# Supplementary material for: Association of Adverse Childhood Experiences Including Low Household Income and Peer Isolation With Obesity Among Japanese Adolescents: Results From A-CHILD Study
Source: Front Public Health. 2022 Apr 5;10:754765. doi: 10.3389/fpubh.2022.754765 (PMC9037323; doi:10.3389/fpubh.2022.754765)
Supplement: Supplementary file 1 [file Data_Sheet_1.pdf]

Supplementary Material

Supplementary Table 1 Relationships between categories of adverse childhood experiences (ACEs)

	Total		4th		6th		8th		N and Percent (%) exposed to another ACEs							
	n	%	n	%	n	%	n	%	1	2	3	4	5	6	7	8
1. Single parenthood	1026	14.8	640	13.4	158	15.4	228	20.2		117 (11.4)	126 (12.7)	50 (5.0)	31 (3.1)	109 (10.9)	155 (15.6)	509 (61.8)
2. Parental history of psychiatric disorders	517	7.4	367	7.7	61	5.9	89	7.9	117 (22.6)		71 (13.9)	49 (9.6)	42 (8.2)	65 (12.7)	75 (14.8)	105 (22.8)
3. Physical abuse from parents (hit, slap)	643	9.3	481	10.0	82	8.0	80	7.1	126 (19.6)	71 (11.0)		128 (20.0)	78 (12.2)	186 (29.0)	100 (16.1)	112 (20.5)
4. Psychological abuse from parents (verb)	306	4.4	224	4.7	41	4.0	41	3.6	50 (16.3)	49 (16.0)	128 (42.0)		69 (22.6)	89 (29.1)	64 (21.4)	45 (16.7)
5. Witness of domestic violence between parents	301	4.3	206	4.3	48	4.7	47	4.2	31 (10.3)	42 (14.0)	78 (25.9)	69 (22.9)		62 (20.6)	48 (16.3)	45 (17.3)
6. Neglect from parents (out, unfed)	562	8.1	438	9.1	56	5.4	68	6.0	109 (19.4)	65 (11.6)	186 (33.3)	89 (15.9)	62 (11.1)		94 (17.1)	93 (19.2)
7. Peer isolation	931	13.4	731	15.3	101	9.8	99	8.8	155 (16.7)	75 (8.1)	100 (10.9)	64 (7.0)	48 (5.2)	94 (10.3)		130 (16.1)
8. Low household income (<3,000,000)	781	11.2	516	10.8	120	11.7	145	12.9	509 (65.2)	105 (13.4)	112 (14.7)	45 (5.9)	45 (5.9)	93 (12.2)	130 (17.1)	

Supplementary Table 2 Distribution of Body Mass Index (BMI) category (N=6946)

	Total		Grade					
			4th (N=4790)		6th (N=1028)		8th (N=1128)	
	N	%	N	%	N	%	N	%
BMI category								
Normal (－1SD-<1SD)	4438	63.9	3053	63.7	621	60.4	764	67.7
Underweight (<－1SD)	1212	17.4	778	16.2	232	22.6	202	17.9
Overweight (1SD<－2SD)	928	13.4	685	14.3	123	12.0	120	10.6
Obesity (2SD+)	368	5.3	274	5.7	52	5.1	42	3.7

Supplementary Table 3 Correlation among characteristics, exposure, and outcome variables

	Correlation coefficient (r)								
	1	2	3	4	5	6	7	8	9
1. Child’s sex									
2. Grade	0.02								
3. Maternal age	0.005	0.18**							
4. ACE total score	-0.13**	-0.07**	-0.08**						
5. Child’s BMI category	-0.09**	-0.04**	0.01	0.05**					
6. Maternal BMI category	-0.01	0.01	0.09**	0.05**	0.12**				
7. Paternal BMI category	-0.01	0.02	0.01	0.02	0.10**	0.13**			
8. Screen time	-0.06**	0.15**	0.03*	0.02	0.002	0.02	0.02		

9. Physical activity	-0.08**	-0.14**	-0.03*	-0.07**	-0.04**	-0.02	-0.01	0.003	
10. Having breakfast	0.04*	0.09**	-0.05**	0.09**	0.06**	0.03**	0.02	0.04**	-0.08**

ACE=adverse childhood experience, BMI=body mass index.

Supplementary Table 4 Results of linear regression to examine the associations between ACEs and BMI z score (n=6946)

		Crude	Model 1	Model 2
		β (95% CI)	β (95% CI)	β (95% CI)
ACE total score (0-8)	0	Ref	Ref	Ref
	1	0.04 (−0.4 to 0.12)	0.02 (−0.05 to 0.11)	0.02 (−0.05 to 0.10)
	2	**0.13 (0.04 to 0.22)**	0.07 (−0.02 to 0.16)	0.07 (−0.02 to 0.16)
	3+	**0.12 (0.01 to 0.24)**	0.07 (−0.05 to 0.18)	0.05 (−0.06 to 0.17)
Single parenthood	No	Ref	Ref	Ref
	Yes	**0.15 (0.07 to 0.24)**	**0.14 (0.05 to 0.23)**	**0.16 (0.07 to 0.25)**
Parental history of psychiatric disorders	No	Ref	Ref	Ref
	Yes	0.01 (−0.10 to 0.12)	0.01 (−0.10 to 0.12)	−0.01 (−0.11 to 0.10)
Physical abuse from parents (hit, slap)	No	Ref	Ref	Ref
	Yes	0.06 (−0.04 to 0.17)	0.03 (−0.08 to 0.13)	0.03 (−0.08 to 0.13)
Psychological abuse from parents (verb)	No	Ref	Ref	Ref
	Yes	−0.02 (−0.16 to 0.12)	−0.04 (−0.19 to 0.10)	−0.07 (−0.21 to 0.07)

Witness of domestic violence between parents	No	Ref	Ref	Ref
	Yes	−0.01 (−0.16 to 0.13)	0.003 (−0.14 to 0.15)	−0.01 (−0.15 to 0.13)
Neglect from parents (out, unfed)	No	Ref	Ref	Ref
	Yes	−0.03 (−0.13 to 0.07)	−0.06 (−0.16 to 0.04)	−0.06 (−0.16 to 0.04)
Peer isolation	No	Ref	Ref	Ref
	Yes	**0.13 (0.04 to 0.22)**	0.06 (−0.03 to 0.15)	0.06 (−0.03 to 0.15)
Low household income (<3,000,000)	No	Ref	Ref	Ref
	Yes	**0.14 (0.05 to 0.24)**	**0.14 (0.04 to 0.24)**	**0.15 (0.05 to 0.25)**

Note. All analyses were weighted for grade. Model 1 included maternal age, child's sex, grade, year conducted a survey, and schools in Adachi City. Model 2 added paternal obesity into Model 1.

ACE=adverse childhood experience, BMI=body mass index, RRR=relative risk ratio, 95%CI=95% confidence interval.
